# On the origin of giant seeds: the macroevolution of the double coconut (*Lodoicea maldivica*) and its relatives (Borasseae, Arecaceae)

**DOI:** 10.1111/nph.16750

**Published:** 2020-07-29

**Authors:** Sidonie Bellot, Ross P. Bayton, Thomas L. P. Couvreur, Steven Dodsworth, Wolf L. Eiserhardt, Maïté S. Guignard, Hugh W. Pritchard, Lucy Roberts, Peter E. Toorop, William J. Baker

**Affiliations:** ^1^ Royal Botanic Gardens, Kew Richmond, Surrey TW9 3AE UK; ^2^ Department of Biological Sciences University of Reading Whiteknights PO Box 217 Reading, Berkshire RG6 6AH UK; ^3^ DIADE, IRD University of Montpellier 911 Avenue Agropolis Montpellier 34394 France; ^4^ School of Life Sciences University of Bedfordshire Luton LU1 3JU UK; ^5^ Department of Biology Aarhus University Ny Munkegade 116 Aarhus C 8000 Denmark; ^6^ Royal Botanic Gardens, Kew Wakehurst Place, Wellcome Trust Millennium Building Ardingly West Sussex RH17 6TN UK; ^7^ Department of Zoology University of Cambridge Downing Street Cambridge CB2 3EJ UK

**Keywords:** Arecaceae, biogeography, coco de mer, Coryphoideae, megafauna, phylogenetics, seed dispersal, seed size

## Abstract

- Seed size shapes plant evolution and ecosystems, and may be driven by plant size and architecture, dispersers, habitat and insularity. How these factors influence the evolution of giant seeds is unclear, as are the rate of evolution and the biogeographical consequences of giant seeds.
- We generated DNA and seed size data for the palm tribe Borasseae (Arecaceae) and its relatives, which show a wide diversity in seed size and include the double coconut (*Lodoicea maldivica*), the largest seed in the world. We inferred their phylogeny, dispersal history and rates of change in seed size, and evaluated the possible influence of plant size, inflorescence branching, habitat and insularity on these changes.
- Large seeds were involved in 10 oceanic dispersals. Following theoretical predictions, we found that: taller plants with fewer‐branched inflorescences produced larger seeds; seed size tended to evolve faster on islands (except Madagascar); and seeds of shade‐loving Borasseae tended to be larger.
- Plant size and inflorescence branching may constrain seed size in Borasseae and their relatives. The possible roles of insularity, habitat and dispersers are difficult to disentangle. Evolutionary contingencies better explain the gigantism of the double coconut than unusually high rates of seed size increase.

## Introduction

Seed size is a major determinant of seed dispersal and seedling establishment (Moles, 2018). Knowing the rates and drivers of seed size evolution is therefore essential to improve predictions of species' responses to changing ecological conditions. Seed size varies by orders of magnitude in almost all angiosperm orders (Linkies *et al*., 2010). This lability has been related to many factors, such as disperser availability, plant size and habitat shadiness (Willson & Traveset, 2010; Leishman *et al*., 2000; Moles *et al*., 2006). The challenge of disentangling these factors impedes understanding of seed size variation within lineages and is an obstacle to a predictive understanding of seed size evolution. The evolution of extreme seed sizes, which define the boundaries of the global seed size distribution, remains particularly unclear.

**Fig. 1:**
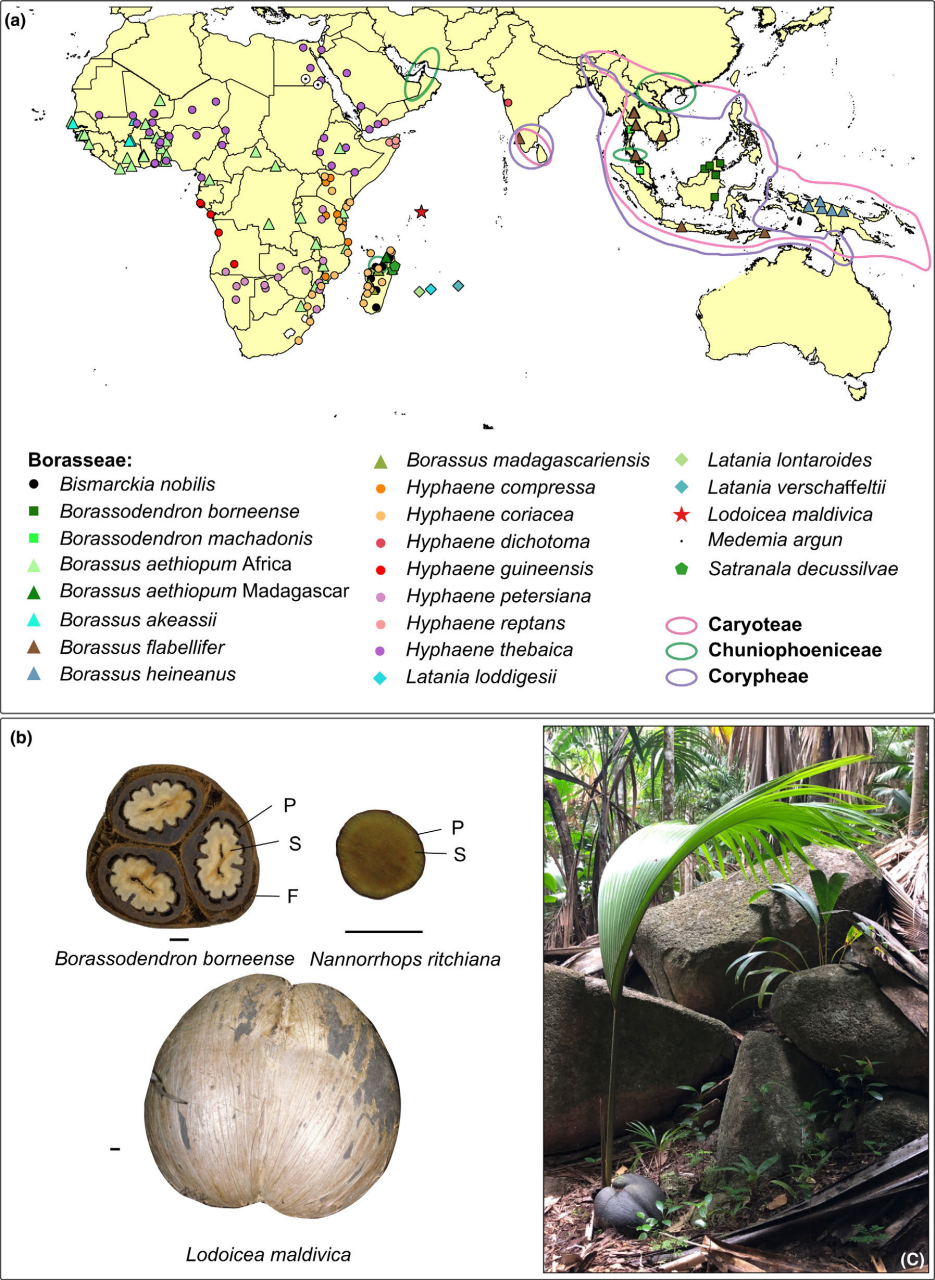
Distribution and seed morphology of selected lineages in the syncarpous clade. (a) Distribution of the syncarpous tribes and of Borasseae species. (b) Examples of fruit, pyrene and seeds of Chuniophoeniceae and Borasseae. I, *Lodoicea maldivica* (picture by J. Dransfield); II, *Borassodendron borneense* J.Dransf. (picture from https://plants.jstor.org); III, *Nannorrhops ritchieana* (Griff.) Aitch. (picture by S. Bellot). F, fruit; P, pyrene; S, seed. Bar, 1 cm. (c) *L. maldivica* germinating in its native habitat in the Seychelles (picture by D. Gower).

One of the most extreme examples of seed size variation is found in the ‘syncarpous clade’ of the fan palm subfamily Coryphoideae (Arecaceae). This clade consists of 16 genera in four tribes restricted to the Old World tropics (Dransfield *et al*., 2008; Baker & Dransfield, 2016): Borasseae, Corypheae, Caryoteae and Chuniophoeniceae, and is supported by all taxa having a syncarpous gynoecium (Rudall *et al*., 2011) and by molecular studies (Baker *et al*., 2009; Barrett *et al*., 2016). Species of the syncarpous clade occur in diverse habitats (dry or wet, open or forested) on islands and continents (Fig. 1a). Seed length in the clade varies from a few millimetres (Caryoteae, Chuniophoeniceae and Corypheae) up to several centimetres (Borasseae) and culminates in the Seychelles endemic *Lodoicea maldivica* (J.F.Gmel.) Pers. that produces the famous ‘double coconut’ or ‘coco de mer’ (Fig. 1b,c), the world's largest seed, which can reach almost 0.5 m in length (17–48 cm according to Morgan *et al*. (2017a)) and is said to weigh up to 25 kg (Tomlinson, 2006; Dransfield *et al*., 2008). The double coconut belongs to the large‐seeded tribe Borasseae, suggesting that the drivers of its large size may also have acted on the tribe's ancestors. Thus, the giant seed size of *Lodoicea* must be explored within the context of Borasseae and the broader syncarpous clade.

The factors responsible for seed size diversity in the syncarpous clade have never been investigated. The large seeds (> 4 cm) of some Borasseae species are assumed to be an adaptation to dispersal by megafauna (> 1000 kg) (Guimarães *et al*., 2008; Onstein *et al*., 2018), and this is supported by their current dispersal by large mammals as reviewed in Zona & Henderson (1989) (see also Discussion section). However, the availability of large dispersers does not explain differences in seed size among the large‐seeded species or between them and co‐occurring species with smaller seeds, and it is well known that large animals also disperse small seeds (Chen & Moles, 2015). A previous study (Edwards *et al*., 2002) suggested that the giant seed of *Lodoicea* could result from it lacking a dispersal agent and/or from increased forest cover, but other Borasseae were not considered. Occurring only in the Seychelles, *Lodoicea* is considered to be a case of island gigantism, but it is unknown if insularity promoted seed size change in this lineage and in its island relatives in Madagascar, New Guinea and the Mascarenes (Fig. 1a). Seed size is likely to have also influenced the dispersal of Borasseae around the Indian Ocean, but this has not been investigated formally. A positive correlation between seed mass and seed dispersal distance has been demonstrated (Thomson *et al*., 2011; Onstein *et al*., 2019). However, this has not been tested for the much larger seeds of Borasseae, which are all dispersed by animals (except for *Lodoicea*, which has no known disperser) and appear not to survive dispersal by floating in sea water (Gunn & Dennis, 1976; Zona & Henderson, 1989).

There are at least six published hypotheses that could explain the seed size of *Lodoicea* and/or seed size change in the syncarpous clade. Although certainly not exhaustive, these hypotheses can be evaluated because clade‐wide data are available. The allometry hypothesis (H1) stipulates that change in plant size will trigger seed size change in the same direction, due to physiological and/or physical constraints (Moles *et al*., 2005a, 2006; Rees & Venable, 2007). Related to H1, Corner's (1949) rule of axial conformity provides a further hypothesis (H2) that less‐branched axes can carry larger appendages (Tomlinson, 2006), in this instance larger fruits with the ability to contain larger seeds. The dispersal agent hypothesis (H3), which is a generalisation of the megafauna idea, stipulates that change in dispersal agent size will trigger seed size change in the same direction, to maximise dispersal (Eriksson, 2008; Galetti *et al*., 2013, 2018). The sibling competition hypothesis (H4) proposes that loss of dispersal agent will result in fewer seeds and a seed size increase, to avoid competition under the mother tree (Edwards *et al*., 2002). The island syndrome hypothesis (H5), which is a generalisation of the island gigantism idea, specifies that arrival on an island leads to increases or decreases in seed size, in response to new selective pressures (Carlquist, 1974; Kavanagh & Burns, 2014; Biddick *et al*., 2019). Finally, the shade hypothesis (H6) states that increase or decrease in habitat shadiness will trigger seed size increase or decrease, in response to lower or higher light availability for the seedling (Leishman *et al*., 2000; Edwards *et al*., 2002). These possible drivers of seed size evolution are not mutually exclusive and may be acting in concert, for instance if disperser availability changes because of changes in habitat.

Hypotheses such as these that might explain the evolution of the extraordinary double coconut have never been formally tested, either separately or in relation to each other. To achieve this, a well supported phylogeny of the double coconut and its relatives is required, as well as supporting data from key characters and environmental variables. Borasseae and the syncarpous clade have never been studied phylogenetically beyond genus‐level and synthetic analyses (Baker *et al*., 2009; Faurby *et al*., 2016). Seed size data are incomplete for palms, notwithstanding a recent trait dataset for the family (Kissling *et al*., 2019). These knowledge gaps must be filled in order to understand how Borasseae achieved their current distributions and seed sizes, and to clarify the origin of the double coconut.

Here, we present a macroevolutionary study of the biogeography and possible drivers that led to the origin of the double coconut. First, we inferred a new phylogeny of tribe Borasseae (including the double coconut) in the broader context of the syncarpous clade based on novel DNA sequence data from two nuclear genes and five plastid regions. Second, we completed molecular dating and ancestral range inferences to establish a biogeographic framework and examine the frequency and age of oceanic dispersals. Thirdly, we generated a novel seed trait dataset from which ancestral seed sizes were estimated and their variation correlated with changes in key variables (plant size, architecture, insularity and habitat). We then discussed our results in the context of the six hypotheses outlined above. In this way, we lay the foundations for future ecological and evolutionary research to address one of the most remarkable phenomena in the plant kingdom.

## Materials and Methods

### Taxon sampling, DNA extraction, PCR amplification and sequencing

We sequenced the DNA of 60 palm specimens, representing all palm subfamilies, all tribes of subfamily Coryphoideae, all genera of the syncarpous clade and 80% of Borasseae species (Supporting Information Table S1). Seven DNA loci were chosen for phylogenetic inference from among regions that had already proven to be informative for palms (Baker *et al*., 2009). The selected regions comprised two low‐copy nuclear regions, *prk* (entire intron 4, partial exons 4 and 5) and *rpb*2 (intron 23), and five plastid regions: the intergenic spacers *atp*B–*rbc*L, *trn*D–*trn*T and *trn*L–*trn*F, the intron of *rps*16 and the protein‐coding gene *rbc*L.

Total genomic DNA was extracted from fresh or silica‐dried leaf material using a CTAB protocol modified from Doyle & Doyle (1990) or a DNeasy Plant Mini Kit (Qiagen, Crawley, West Sussex, UK). Extracted DNAs were cleaned and concentrated using the QIAquick PCR Purification Kit (Qiagen). PCR and sequencing of *atp*B–*rbc*L, *rbc*L, *rps*16, *trn*D–*trn*T and *trn*L–*trn*F followed Demesure *et al*. (1995), Asmussen & Chase (2001), Oxelman *et al*. (1997), Manen *et al*. (1994) and Taberlet *et al*. (1991) respectively. For the nuclear genes, primers published by Lewis & Doyle (2002) and Roncal *et al*. (2005) were used following a PCR protocol set out by Norup *et al*. (2006). PCR products were purified using the QIAquick PCR Purification Kit (Qiagen). Cycle‐sequencing protocols are described in Norup *et al*. (2006). Cleaned cycle‐sequencing products were sequenced using either an ABI Prism 377 or an ABI Prism 3100 automated sequencer (Applied Biosystems, Foster City, CA, USA) according to the manufacturer's protocols. Sequences were submitted to the GenBank database (see accession numbers in Table S1).

### Sequence alignment and phylogenetic analyses

Sequences were checked using sequencher 4.1.2 (Gene Codes, Ann Arbor, MI, USA), and aligned using the ClustalW algorithm implemented in megalign 5.00 (Clewley & Arnold, 1997). Individual alignments were concatenated in a supermatrix totalling 7311 nucleotide sites.

A maximum likelihood (ML) phylogenetic analysis was performed for each DNA region separately, using raxml 8.2.10 (Stamatakis, 2014) with a GTR + G model of evolution and 500 bootstrap replicates. The most likely trees were rooted using *Calamus aruensis* (Calamoideae) as the outgroup and then compared. Some conflicts supported by more than 70% of the bootstrap replicates could be identified but running downstream analyses after excluding sequences of taxa involved in these conflicts resulted in the same topology and similar ages, so we present only results based on all sequences.

To obtain a ML phylogeny, the concatenated matrix was partitioned following the best scheme of evolutionary models found by partitionfinder2 (Lanfear *et al*., 2017) and analysed with raxml 8.2.10 (Stamatakis, 2014) including 1000 bootstrap replicates.

### Molecular dating and biogeography

Ages of divergence were estimated using Beast 1.8.4 (Drummond *et al*., 2012). We used a GTR + G model with four rate categories and an uncorrelated lognormal relaxed clock to model the nucleotide substitution rate. A birth–death model with incomplete sampling (Stadler, 2009) was used as the speciation prior after that path‐sampling estimations of marginal likelihoods and Bayes factors calculations showed strong evidence (Kass & Raftery, 1995) for this prior over a Yule prior. The inference was run independently two times for 50 million generations, resulting in effective sampling sizes > 200 for all parameters and convergence towards the same posterior distributions (visualised using tracer v.1.7 software; Rambaut *et al*., 2018). A burn‐in fraction of 25% of the trees was discarded before reporting median posterior ages on the maximum clade credibility tree using tree annotator v.1.8.4 (Drummond *et al*., 2012). Absolute ages were obtained by calibrating the clock with three fossils. The first fossil, *Sabalites carolinensis* (Berry, 1914) characterises the stem of Coryphoideae, and was found in the Black Creek formation, near Langley, Aiken County, South Carolina, USA, and dated from the Santonian (83.4–86.8 million years ago (Ma)) (Iles *et al*., 2015) to the late Coniacian age (86.8–90.1 Ma) (Berry, 1914), so we calibrated the crown node of the clade formed by Ceroxyloideae, Coryphoideae and Arecoideae with a prior age following an exponential distribution of mean 5 Ma, with an offset of 83 Ma. The second fossil has been reliably attributed to Sabaleae (Manchester *et al*., 2010; Cano, 2018), and was found in the Aguja formation of the Big Bend National Park, Texas, USA, and dated to the Campanian age (71.9–83.8 Ma), so we calibrated the crown node *Sabal*‐*Coccothrinax* with a prior age following an exponential distribution of mean 5 Ma, with an offset of 72 Ma, to take into account the possibility that the fossil would be more recent than the divergence of Sabaleae and *Coccothrinax*. The third fossil, *Hyphaenocarpon indicum* (Matsunaga *et al*., 2019), characterises the crown of Hyphaeninae, was found in the Deccan Intertrappean Beds of the Shahpura Mandla District, Madhya Pradesh, India, and dated to the Late Maastrichtian to early Danian (64–67 Ma), so we calibrated the crown node of Hyphaeninae with a prior age following an exponential distribution of mean 5 Ma, with an offset of 64 Ma.

We inferred ancestral ranges using biogeoBears (Matzke, 2013). As summarised in Table S2, seven areas were defined based on current and past geographic isolation, and the present occurrence of each taxon in those areas was recorded using the *World Checklist of Selected Plant Families* (WCSP, 2017). We compared Dec, Bayesarealike and Divalike models, including or not founder‐event speciation (j parameter; Matzke, 2014), and including or not the possibility for a range out of the specified areas (‘null range’; Massana *et al*., 2015).

### Plant size, inflorescence branching and habitat data

To investigate their relationship with seed size, plant maximum reported height and inflorescence branching order were recorded from the literature (Table S2). To characterise the habitat of each species, their distributions were assessed using georeferenced data from Gbif (https://www.gbif.org/occurrence/search?taxon_key=7681, accessed on 14 November 2016), Rainbio (Dauby *et al*., 2016; Sosef *et al*., 2017), the herbarium of the Royal Botanic Gardens, Kew, and the literature (Henderson, 2009; Stauffer *et al*., 2014). Data were cleaned to exclude occurrences out of the native range of the species (based on the *World Checklist of Selected Plant Families* (WCSP, 2017) and *Palmweb* (Palmweb, 2019)), except for *Hyphaene petersiana*, for which a few points in neighbouring countries of its known native range were kept because they represented plausible occurrences and did not change habitat inferences. One occurrence per grid cell of the MODIS land cover dataset (Friedl *et al*., 2010; Channan *et al*., 2014) was then sampled for each species, and the habitat of each occurrence was defined as closed if the land cover was a type of forest (values 1–6) or otherwise open (values 7–14). Species with more than 50% of occurrences falling in a given habitat (closed or open) were classified as occurring in this habitat, whereas three species with exactly 50% of occurrences in each habitat were classified as occurring in a closed habitat, based on the literature (Table S2).

### Seed data and ancestral state estimations

To obtain robust and comprehensive data on seed size variation in Borasseae and other syncarpous tribes, we analysed specimens in the collections of the Royal Botanic Gardens, Kew (herbarium and the Millennium Seed Bank), and data from the literature. Borasseae fruits contain one to three seeds, and each seed is surrounded by an endocarp, which can be very thick (Fig. 1b). These endocarps protect the seed when it is dispersed, and thus the seed plus its endocarp, hereafter termed the pyrene, is the relevant unit to consider for questions related to dispersal. Up to 10 collections per species were sampled, from which the length of up to 10 fruits and of their pyrenes and seeds were measured using a set of digital callipers. Table S3 summarises the number of collections and fruits, pyrenes and seeds measured per species. When possible, we calculated for each collection the ratios of fruit length and pyrene length, fruit length and seed length, and pyrene length and seed length. To increase our seed and pyrene datasets, we then used those ratios to estimate seed or pyrene length from other collections of the same species that had only fruits and/or pyrenes available to measure (Table S3). Fruit, pyrene and seed width and height were also collected and used to estimate volumes, but as our results were qualitatively identical to these obtained using only lengths (not shown) and as most studies on seed size rely only on length, we only used the latter in our final analyses.

Ancestral plant heights, pyrene lengths and seed lengths were estimated using the fastAnc function in the package contmap (Revell, 2012, 2013), which assumes trait evolution under a Brownian motion model. This model had the smallest AICc when fitted to our data, compared with the other models available in the geiger package (Harmon *et al*., 2008). The effects of plant size, inflorescence branching order (low, high), insularity (island, continent), and habitat (open, closed) on seed or pyrene size (log‐transformed to achieve normality and equal variances) were tested by fitting linear models with all variables and their interactions. A stepwise model reduction procedure was used to remove nonsignificant terms and the model with the lowest corrected AIC was selected using the aiccmodavg R package (Mazerolle, 2019). The procedure was repeated with models including phylogenetic structure, using the phylogenetic generalised least squares (pgls) function from the caper R package (Orme *et al*., 2018). All analyses were performed in R 3.5 (R Core Team, 2018), using the packages ape (Popescu *et al*., 2012), ggplot2 (Wickham, 2016), ggpubr (https://rpkgs.datanovia.com/ggpubr/), ggrepel (http://github.com/slowkow/ggrepel), gridextra (https://cran.r‐project.org/web/packages/gridExtra/index.html), phytools (Revell, 2012), and rcolorbrewer (https://CRAN.R‐project.org/package=RColorBrewer). Analyses were repeated without *Lodoicea* to assess its influence on the results.

### Data availability

The DNA sequence data underlying this study are available in the GenBank database at https://www.ncbi.nlm.nih.gov/genbank/ (accession numbers in Table S1) and the morphological measurements and collection information are available in Table S3.

## Results

### Phylogenetic relationships in the syncarpous clade are largely resolved

**Fig. 2:**
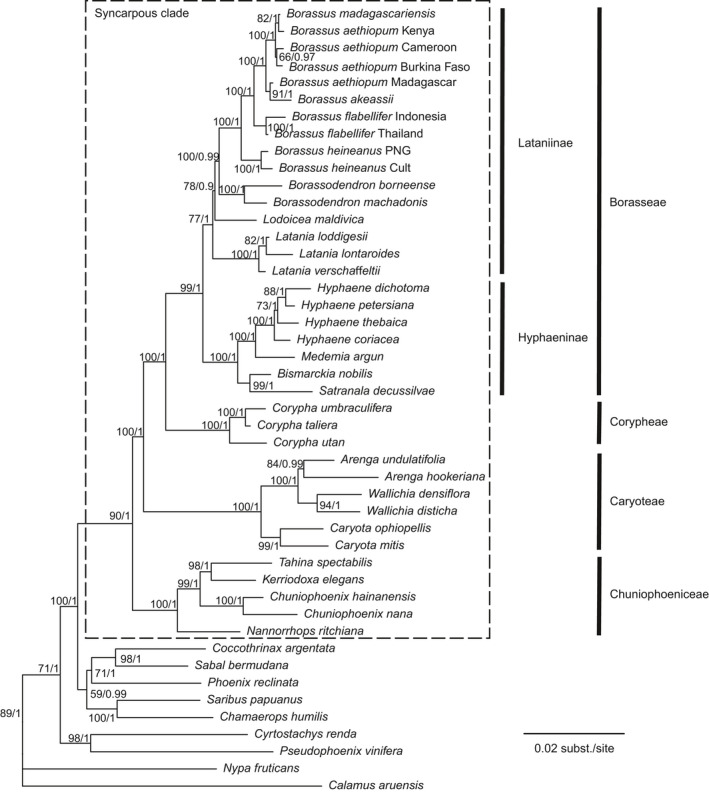
Phylogeny of the syncarpous clade obtained by maximum likelihood analysis of two nuclear and three plastid regions. Numbers at nodes are bootstrap support percentages and Bayesian posterior probabilities (the latter obtained from the Bayesian analysis of the same dataset).

The syncarpous clade, the four tribes that constitute it, and all their genera were resolved as monophyletic, and identical relationships were recovered by the ML (Fig. 2) and Bayesian (Fig. S1) inferences, with bootstrap supports and posterior probabilities generally ≥ 90% and ≥ 0.97 respectively (Fig. 2). Borasseae and Corypheae were recovered as sister tribes and were more closely related to Caryoteae than to Chuniophoeniceae. In the latter, *Tahina* was sister to *Kerriodoxa*, and *Chuniophoenix* sister to this clade (but see the Discussion section). In Caryoteae, *Wallichia* and *Arenga* were sister taxa and in Corypheae, *C. umbraculifera* was sister to *C. taliera*. In borassoid subtribe Hyphaeninae, two sister pairs were resolved, the first comprising *Bismarckia* and *Satranala*, the second *Hyphaene* and *Medemia*. *Hyphaene dichotoma* and *H. petersiana* were sister species and more closely related to *H. thebaica* than to *H. coriacea*. In borassoid subtribe Lataniinae, *Borassodendron* and *Borassus* were sister genera, and this clade in turn was sister to *Lodoicea*. The position of *Lodoicea* was, however, only supported by 78% bootstrap support and a posterior probability of 0.9, reflecting the short branches that separated Lataniinae ancestors from one another (Fig. 2). African *Borassus* formed a clade more closely related to the Indo‐Asian *B. flabellifer* than to the New Guinean *B. heineanus*. *Borassus aethiopum* was recovered as paraphyletic, with accessions from Burkina Faso, Cameroon, Kenya and Madagascar intermixed with *B. madagascariensis* (from Madagascar) and *B. akeassii* (from West Africa; Fig. 2).

### Borasseae dispersed across oceans and long distances multiple times

**Fig. 3:**
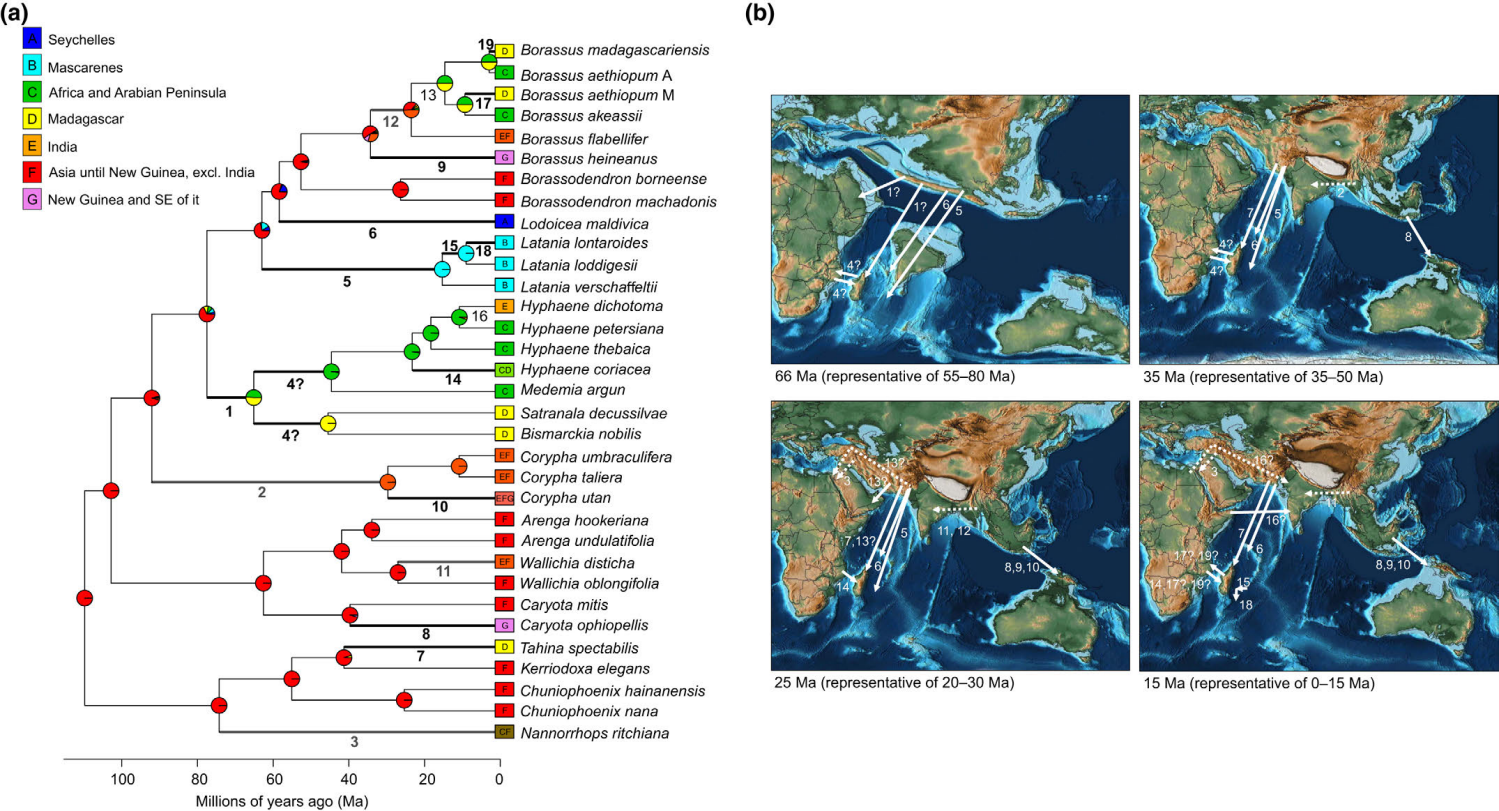
Biogeography of the syncarpous clade. (a) Ancestral range probabilities obtained using the Bayesarealike model including founder‐event speciation and excluding the possibility of a ‘null’ range implemented in BioGeoBears. Numbers on branches refer to the dispersal events illustrated in (b), with continental dispersals represented by numbers and branches in bold grey, oceanic dispersals in bold black, and ambiguous dispersals in regular black. (b) Approximate location and direction of dispersal events. Numbers refer to the events indicated on (a). When a dispersal could have happened during multiple time slices it is indicated on all of them. Dashed lines, continental dispersals; full lines, oceanic dispersals. Question marks indicate uncertainty regarding what lineage dispersed. Maps obtained from the PALEOMAP project (Scotese, 2016).

According to the selected biogeographic model (see Notes S1 for the rationale behind model selection, and Fig. S2 for results with alternative models), the ancestors of the syncarpous clade were most likely to have occurred in Asia 110 Ma (see Fig. S1 for 95% highest posterior densities of ages), from where they dispersed multiple times around the Indian Ocean (Fig. 3a,b). A minimum of 19 dispersals was required to achieve this distribution: at least four dispersals over land (one in each syncarpous tribe), at least 13 dispersals over sea (10 in Borasseae and one in each other tribe), and two uncertain dispersals (in Borasseae). The spatio‐temporal location and the direction of these events is described below.

Tribes Caryoteae, Chuniophoeniceae and Corypheae diversified mostly in Asia and experienced at least six dispersals. *Corypha* ancestors reached India by land between 55 Ma, when both continents became connected (Fig. 3b), and 30 Ma, when the current *Corypha* lineages started to diversify. The ancestors of *Nannorrhops* are likely to have expanded their range by land into the Arabic Peninsula by 30 Ma, when it became connected to Asia (Fig. 3b). These lineages might also have dispersed as early as 92 Ma and 74 Ma, respectively, when they diverged from their sister lineages, but this would have required unlikely 40 Ma‐lasting transoceanic gene flows. The ancestor of *Tahina* crossed the Indian Ocean to disperse from Asia to Madagascar by 41 Ma, and the ancestors of *Caryota ophiopellis* and *Corypha utan* both dispersed across seas from Asia to Vanuatu and New Guinea 40 Ma and 30 Ma onwards respectively (Fig. 3b). Finally, *Wallichia disticha* expanded its range by land from Asia to India by 24 Ma.

Borasseae experienced a minimum of 12 dispersals. The first one happened when the ancestor of Hyphaeninae crossed the Indian Ocean to go from Asia to Africa or Madagascar 77–65 Ma (Fig. 3b). This was followed at the latest 45 Ma by a second oceanic dispersal between Madagascar and Africa, the direction of which remains unclear. Then, the *Hyphaene* + *Medemia* clade mostly diversified in Africa with two exceptions: the ancestor of *H. coriacea* crossed the Mozambique Channel and reached Madagascar by 23 Ma and the ancestor of *H. dichotoma* dispersed to India by 11 Ma, either by crossing the Indian Ocean or by land through the Middle East (Fig. 3b). The ancestors of Lataniinae dispersed from Asia to the Mascarenes and the Seychelles in two independent, most likely oceanic, dispersals that occurred by 63 Ma and 58 Ma, and respectively gave rise to *Latania* and *Lodoicea* (Fig. 3b). After reaching the Mascarenes, *Latania* dispersed from Rodrigues to Mauritius by 14 Ma, and then from there to La Reunion by 7 Ma. *Borassodendron* apparently diversified only in Asia 26 Ma, and the ancestor of *Borassus* was also Asian (Fig. 3a). The latter dispersed across seas to New Guinea by 33 Ma, and then the most recent common ancestor (MRCA) of *B. flabellifer* and African *Borassus* expanded by land to India from 24 Ma. Finally, the MRCA of African *Borassus* dispersed to Madagascar or Africa from 15 Ma, by sea or possibly by land via the Middle East (Fig. 3b). Two oceanic dispersal events between Madagascar and Africa subsequently happened, leading to the divergence of *B. akeassii* and the Madagascan *B. aethiopum* 9 Ma, and to the divergence of the African *B. aethiopum* and *B. madagascariensis* 3 Ma. It was not possible to infer the direction of these dispersals.

### Large pyrenes crossed oceans in the ancestors of Borasseae

**Fig. 4:**
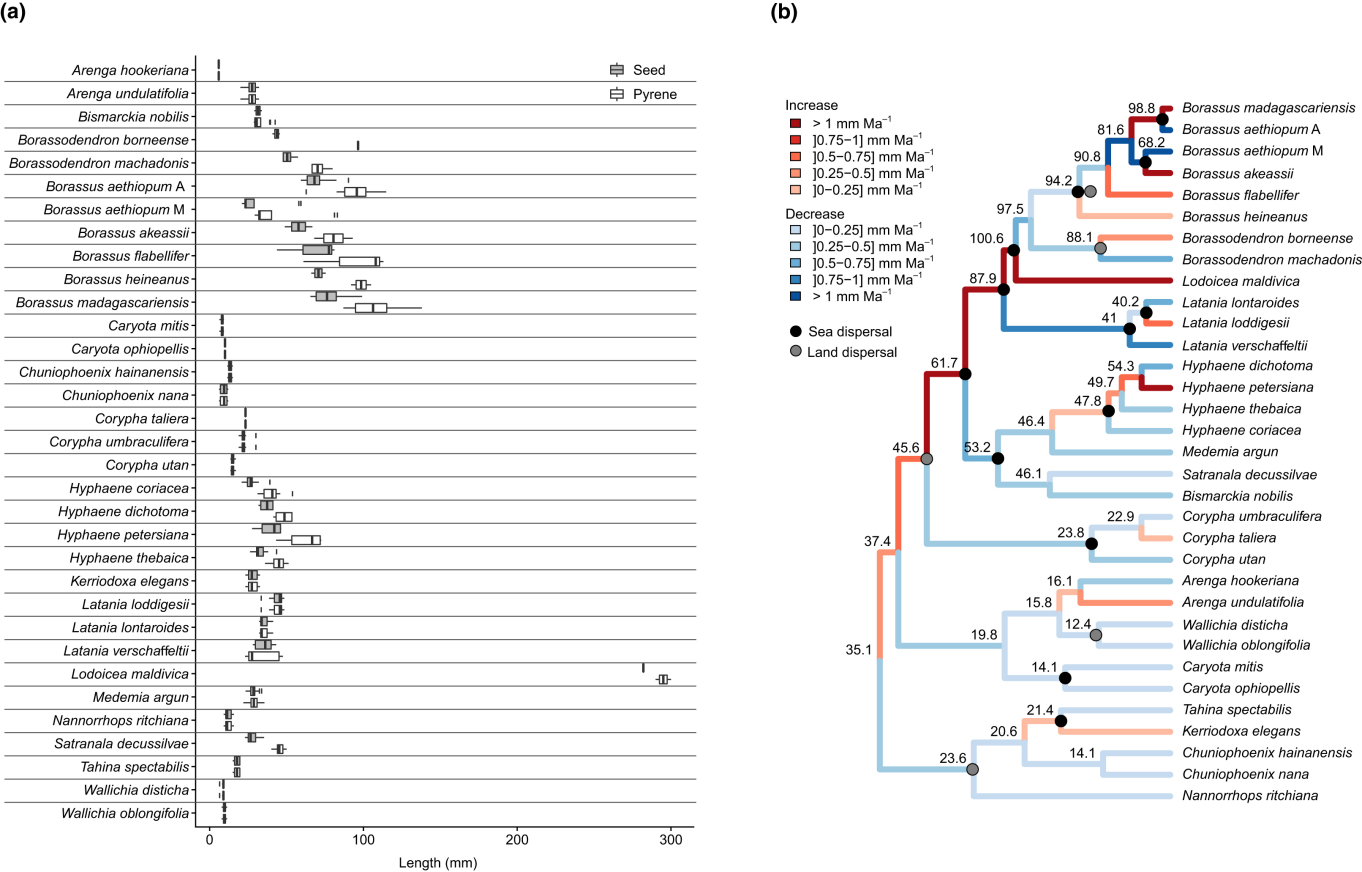
Seed and pyrene sizes in the syncarpous clade. (a) Current seed and pyrene sizes. In the boxplots, the bold line represents the median value, the box spans values from the first to the third quartile, and the lines outside the box extend until the smallest and largest values respectively, no further than 1.5 times the distance between the first and third quartiles. (b) Ancestral pyrene sizes and rates of change in mm Ma^−1^ obtained when including *Lodoicea* in the analyses.

**Fig. 5:**
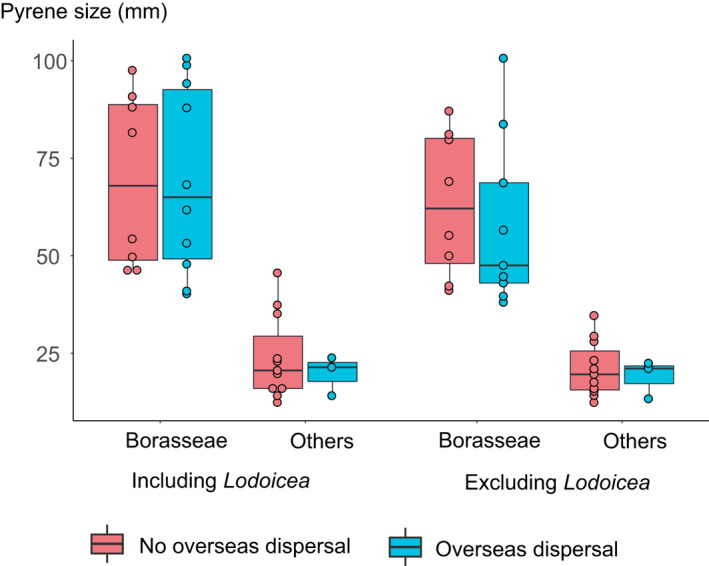
Relationship between pyrene size and oceanic dispersal for the ancestors of the current syncarpous clade species. In the boxplots, circles represent all the data points, the bold line represents the median value, the box spans values from the first to the third quartile, and the lines outside the box extend until the smallest and largest values respectively, no further than 1.5 times the distance between the first and third quartiles.

Median seed and pyrene lengths were similar within species of the syncarpous clade, except in *Borassodendron*, *Borassus*, *Hyphaene petersiana* and *Satranala*, where they differed by 9–53 mm (Fig. 4a). The pyrene, and not the seed, is the dispersing unit (see the Materials and Methods section), so we mostly present results based on pyrene size. *Lodoicea*'s ancestor was estimated to have pyrenes between 55.3 (Fig. S3a; *Lodoicea* excluded from the analyses) and 100 mm long (Fig. 4b; *Lodoicea* included) by the time it diverged and dispersed to the Seychelles. The ancestors of *B. heineanus* and *B. madagascariensis* had similarly large pyrenes involved in oceanic dispersals, with lengths ≥ 82 mm (Figs 4b, S3). The ancestors with the largest pyrenes involved in continental dispersals were those of *B. flabellifer*, with pyrene lengths ≥ 85 mm (Figs 4b, S3). There was no difference in ancestral pyrene size between oceanic and continental dispersals (Fig. 5).

### Pyrene size increased and decreased multiple times and at varied rates

Change in pyrene size was significantly positively correlated with change in seed size (Pearson's *r* > 0.96, *P*‐value < 2.45e‐37; Fig. S3b), so we only present the former. The ancestor of the syncarpous clade had pyrenes 27–35 mm long and we inferred a minimum of 10 or 8 subsequent size increases and 11 or 13 subsequent size decreases, depending whether *Lodoicea* was included or not in the analyses (Figs 4b, S3a). From the ancestral state, pyrene sizes followed opposite trends at each divergence event, increasing in a lineage while decreasing in its sister (Fig. 4b). This pattern was conserved when excluding *Lodoicea* (Fig. S3a), except for *Borassodendron* + *Borassus* whose ancestors either sustained a continuous increase (excl. *Lodoicea*, Fig. S3a) or decrease (incl. *Lodoicea*, Fig. 4b), depending on the analysis.

Rates of increase were on average higher than rates of decrease, and more variable, but the most extreme rates of increase and decrease were similar in intensity (Fig. S3c). *Lodoicea* had the highest rate of increase with 3.4 mm Ma^−1^ (Fig. 4b) and a 193% increase (3.3% per Ma) compared with its ancestor (Fig. S4). The Madagascan *B. aethiopum* had the highest rate of decrease with 4 mm Ma^−1^ (Fig. 4b) and a 5.8% per Ma decrease compared with its ancestor (Fig. S4b), but *Arenga hookeriana* showed the highest absolute percentage of decrease with 63% (Fig. S4a). Results did not change qualitatively when removing *Lodoicea* from the analyses (right panels on Fig. S4).

### Relationships between seed size and plant size, inflorescence branching, insularity and habitat

**Fig. 6:**
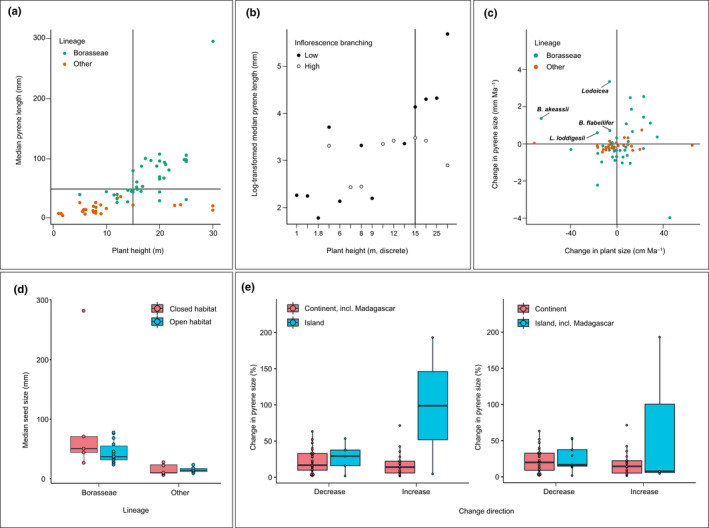
Relationships between plant size, inflorescence branching, insularity, habitat and pyrene or seed size in the syncarpous clade. (a) Ancestral and current plant and pyrene sizes. Vertical and horizontal lines highlight the absence of species smaller than 15 m with pyrenes larger than 40 mm. (b) Interaction plot illustrating how the relationship between plant size (plotted as a discrete variable) and pyrene size depends on inflorescence branching. The vertical line indicates the plant height from which an interaction could clearly be detected. (c) Change in pyrene size and plant size. Vertical and horizontal lines highlight the few lineages with a concomitant decrease in plant height and increase in pyrene size. (d) Comparison of the seed size of current species occurring in closed or open habitats. (e) Comparison of the pyrene size of lineages that dispersed from a continent to an island or not. In the boxplots of panels (d) and (e), circles represent all the data points, the bold line represents the median value, the box spans values from the first to the third quartile, and the lines outside the box extend until the smallest and largest values respectively, no further than 1.5 times the distance between the first and third quartiles.

The allometry and axial conformity hypotheses (H1 and H2) explain size changes in the dispersal unit, that is the pyrene, while the shade hypothesis (H6) explains size changes in the resource‐storing unit, that is the seed, and the island syndrome hypothesis (H5) explains changes in both (see the Introduction section). We focused therefore on both pyrene and seeds to evaluate the evidence supporting these hypotheses. Plotting current and ancestral sizes suggested the existence of a positive linear relationship between plant size and pyrene size for plants < 20 m tall and revealed that all past and present taxa smaller than 15 m had pyrenes shorter than 50 mm (Fig. 6a). Plant size could not explain all the variation observed in pyrene size: some of the tallest taxa had some of the smallest pyrenes, and the pyrene length of *Lodoicea* was approximately three times larger than linearly expected from its height (Fig. 6a). Reflecting this, the best statistical model was the one with pyrene size as a function of the interaction between plant size and inflorescence branching (adjusted *R* ^2^ = 0.6624, *P*‐value = 1.295e‐07; Fig. 6b; Table S4a). There was a significant positive linear relationship between log(pyrene size) and plant size in species with low inflorescence branching (*P*‐value = 0.00025 or 0.00067 without *Lodoicea*; Table S4a,b), but not in species with highly branched inflorescences (*P*‐value = 0.83653 or 0.836567 without *Lodoicea*; Table S4a,b). The effects of plant size and/or inflorescence branching on pyrene size were nonsignificant when accounting for phylogenetic relationships (*P*‐values > 0.05; Table S4c,d). Throughout the evolution of the syncarpous clade, plant size increases were accompanied by both increases or decreases in pyrene size, whereas plant size decreases were mostly accompanied by decreases in pyrene size (Fig. 6c). This was however not the case in the lineages leading to *Lodoicea*, *B. akeassii*, *B. flabellifer* and *L. loddigesii*, where pyrenes became longer despite plants becoming smaller (Fig. 6c).

Borasseae species living in closed habitats tended to have larger seeds than species living in open habitats (even when excluding *Lodoicea*), but this was not the case in the remainder of the syncarpous clade (Fig. 6d). Finally, island lineages tended to show higher rates of change in pyrene size than continental lineages when considering Madagascar as a continent, but not when considering it as an island (Fig. 6e). Statistical tests did not reveal significant effects of habitat or isolation on seed or pyrene size, even in interaction with plant size and inflorescence branching, and best models consistently excluded these two variables (*P*‐values > 0.05; Table S4).

## Discussion

### Oceans are no barrier to dispersal for the world's largest seeds

Our study provided a highly supported hypothesis for the relationships in Borasseae and for their ancestral ranges, allowing us to draw inferences on the evolution and dispersal of the largest‐seeded plants in the world. There were some signs of genetic incongruence in our data (see the Materials and Methods section) and although these did not affect our results, further population and phylogenomic studies in the syncarpous clade are likely to reveal complex past gene flow, especially in Chuniophoeniceae. Such studies may also inform species delimitation, for example, in *Borassus* in which *B. aethiopum* (as defined by Bayton (2007)) was found to be paraphyletic (Fig. 2). Our biogeographic inferences and Borasseae, Caryoteae and Corypheae fossils from UK and India (Harley, 2006; Matsunaga *et al*., 2019) suggest that current syncarpous taxa could be relicts of more diverse and widely distributed lineages. The syncarpous clade is likely to have originated in Asia in the mid‐Cretaceous age, and then, during the Paleogene and the Neogene, recurrently dispersed across land and sea around the Indian Ocean (Fig. 3). These timescales are much older than previously thought (Baker & Couvreur, 2013) due to new fossil evidence in Borasseae (Matsunaga *et al*., 2019). A global revision of palm ages and biogeography will be required to confirm our findings and discuss their implications regarding the evolution of palms and past biomes.

We found that the large seed (and therefore pyrene) size in Borasseae ancestors was no obstacle to recurrent oceanic dispersal events (Fig. 5). These dispersals could have been facilitated by the continuous existence of islands in the Seychelles and Mascarene regions since 70 Ma (Chatterjee *et al*., 2013), and by trade winds from the mid‐Eocene onwards (Samonds *et al*., 2012). Migratory birds, which may have existed since the Cretaceous (Berthold, 1999), could have dispersed the smallest‐seeded syncarpous ancestors. The dispersals of *Borassus* and *H. coriacea* across the Mozambique Channel, of *Borassus* across the Wallace line, and of *Latania* between the Mascarene islands involved pyrenes > 40 mm long (Figs 4b, S3a) that most likely dispersed on floating islands since they cannot germinate after a prolonged exposure to sea water (Gunn & Dennis, 1976). The direction of dispersals across the Mozambique Channel could not be inferred from our results but sea currents of the last 15–20 Ma suggested that the ancestors of African *Borassus* dispersed twice from Madagascar to Africa (Ali & Huber, 2010). Giant *Cylindraspis* tortoises could have dispersed *Latania* between the Mascarene islands, while the giant tortoises (*Aldabrachelys*) that colonised the Seychelles > 23 Ma (Austin & Arnold, 2001; Cheke *et al*., 2017) appear not to have dispersed *Lodoicea* around the archipelago, perhaps because its pyrenes were already too large by then.

### Seed size may not increase at unusually high rates in Borasseae, not even in *Lodoicea*

Unusually high rates of evolution are not required to explain the large seed sizes of Borasseae or even *Lodoicea*. Based on the divergence times inferred here for Borasseae species, only low rates of seed size increase would be required to evolve very large seeds. Few studies have quantified change in seed size over time on macroevolutionary scales (Ackerly, 2009) but one recent study reports changes in the palm *Euterpe edulis* (Galetti *et al*., 2013) that are three orders of magnitude faster than the rate we inferred in *Lodoicea* or other syncarpous lineages (Fig. 4b). Variations in regulatory genes can modify resource accumulation and cell size/number, which could result in relatively quick and large changes in seed size (Linkies *et al*., 2010). The large seeds of Borasseae could therefore have evolved more rapidly than estimated here, in a punctuated manner. Comparing models of gradual or punctuated seed size change informed by plant size, insularity, dispersers or habitat data could elucidate the tempo and drivers of seed size evolution in a statistical framework. This approach could not be used here due to the scarcity of paleo‐ecological data and to the small size of the syncarpous clade.

Uncertainty regarding the mode and tempo of trait evolution is a long‐standing problem in ancestral trait inferences that can be alleviated by incorporating fossils in the analyses (Slater *et al*., 2012). There is only one well identified seed fossil available for Borasseae, and its precise placement remains unclear (Matsunaga *et al*., 2019). This fossil is half of the seed size that we inferred for the MRCA of Borasseae, suggesting that we may have overestimated the seed size of some Borasseae ancestors and underestimated their subsequent rate of increase. We nevertheless refrained from informing our analyses with the size of the fossil because it could represent a derived state only present in some Hyphaeninae (Matsunaga *et al*., 2019).

### Large plants and little‐branched inflorescences allowed larger seeds to evolve

Consistent with the allometry hypothesis (H1), plant size seems to have constrained pyrene (and therefore seed) size in that small plants did not carry large pyrenes (Fig. 6a), and plant size change was enough to explain pyrene size change in many cases (Fig. 6c). In agreement with the axial conformity hypothesis (H2), some large palms such as *Corypha* do not carry large pyrenes, because they evolved highly branched inflorescences (Fig. 6b) that could not support large appendages. Both tendencies have been found in other lineages (Grubb *et al*., 2005; Moles *et al*., 2005b). The combination of large plant size and unbranched or scarcely branched inflorescences may therefore have been decisive in allowing the evolution of large pyrenes in Borasseae. However, some Borasseae, including *Lodoicea*, have smaller or larger pyrenes than predicted by their plant size and inflorescence structure (Fig. 6a,c), suggesting that additional factors drove seed and pyrene size change in these species.

### The roles of dispersal agents, habitat and insularity cannot easily be disentangled

We could not detect a significant effect of habitat and/or insularity on the seed size of Borasseae and their relatives. This result should be interpreted with caution because our sampling was small and unbalanced between categories (Fig. 6d,e). However, it could also be that the effects of habitat or insularity depend on dispersal agent availability and therefore would only be detected when including the latter in the statistical analyses. Palaeontological data are too scarce to formally investigate the role of past dispersers in the evolution of seed size in the syncarpous clade, but comparing our results with available information on past and present dispersers reveals a possibly important role of the latter in interaction with both habitat and insularity.

Today, large primates (*Papio*, *Pongo pygmeus)*, megabats (*Eidolon* and *Pteropus*), elephants (*Loxodonta africana*) and ratites (*Casuarius*) disperse Borasseae (Zona & Henderson, 1989; Pangau‐Adam & Mühlenberg, 2014), allowing them to have large seeds, and possibly selecting for them, in agreement with the dispersal hypothesis (H3). The heaviest Eurasian herbivorous mammal was already 663 kg *c*. 57 Ma (Smith *et al*., 2010) and large flightless birds evolved in the last 25 Ma (Mitchell *et al*., 2014), suggesting that large animals (including megafauna (Guimarães *et al*., 2008)) could also have selected for large‐seeded Borasseae in the past. The increase in pyrene and seed size observed in Borasseae since their divergence from other tribes (Fig. 4b) is consistent with a global increase in seed size observed in angiosperms from the Upper Cretaceous onwards. The latter has been attributed to global faunal and climatic changes that, respectively, may have facilitated the evolution of large seeds (dispersal hypothesis H3) and resulted in more closed habitats where large seeds were advantageous (shade hypothesis H6) (Tiffney, 1984; Eriksson *et al*., 2000; Eriksson, 2008). The co‐occurrence in time and global nature of these changes in fauna and climate makes it difficult to differentiate their respective influences on seed size evolution, in Borasseae as in other angiosperms.

The roles of dispersers and insularity are also difficult to distinguish from each other because islands often have different habitats and smaller guilds of dispersers than continents. Giant tortoises (*Cylindraspis*) were present in the Mascarenes for the past 17–23 Ma (Cheke *et al*., 2017). They have been shown to act as seed dispersers (Griffiths *et al*., 2011) and even smaller tortoises can ingest seeds of the size of *Latania* pyrenes (Jerozolimski *et al*., 2009), so they may have selected for the pyrene size decrease observed in *Latania* after its arrival in the archipelago. Out of the four lineages that arrived in Madagascar (Fig. 3), three decreased in pyrene size (Figs 4b, S3a), suggesting parallel adaptations to a smaller guild of dispersers. This finding is supported by the large herbivorous fauna of Madagascar (comprising giant lemurs and elephant birds) being smaller than the largest Asian or African fauna (Godfrey *et al*., 2008).

### On the origin of the double coconut

The double coconut is one of the most celebrated and mysterious phenomena in the natural world. The evolution of a giant seed on so remote an island poses many enduring questions. How did it get there? Did it occur anywhere else in the past? Why is it so big? What are the consequences of its size? Our study sheds light on some of these questions, which we summarise here.

The ancestors of the double coconut occurred in mainland Asia, were massive and bore large fruits that were likely adapted to dispersal by megafauna (Guimarães *et al*., 2008; Onstein *et al*., 2018). Large fruits could be sustained by these palms on account of their great size and unbranched (or sometimes once‐branched) inflorescences, reflecting Corner's rule (Corner, 1949) that large axes are required to bear large appendages. Thus, these ancestors were well placed to evolve even larger seeds. In the Palaeocene, *Lodoicea*'s ancestors reached the Seychelles, which at that time was a large archipelago influenced by oceanic currents and trade winds. Paradoxically, fresh seeds of modern *Lodoicea* and its relatives do not float and are likely to be killed by long exposure to sea water (Gunn & Dennis, 1976). One or more freak dispersal events are required to explain the double coconut's arrival in the Seychelles. For example, it is possible that ancestral *Lodoicea* dispersed on a floating vegetation mat, which protected it from sea water.

There is no evidence that animals capable of dispersing megafaunal fruits have ever occurred in the Seychelles, except for giant tortoises (*Aldabrachelys*). Our inferences suggest that seeds produced by the *Lodoicea* lineage were already too large to be consumed by these tortoises when the latter arrived in the Seychelles > 23 Ma (Austin & Arnold, 2001; Cheke *et al*., 2017). Lacking a disperser, ancestral *Lodoicea* plants producing fewer seeds were likely more successful than plants producing more seeds that would compete with each other (Edwards *et al*., 2002). Thus, more resources could be allocated to each seed, which, unchecked, led to gigantism. We do not know if *Lodoicea* continues to evolve towards even larger seeds even now, or indeed what the biophysical limits of this phenomenon might be. Other than rolling down hill (Morgan *et al*., 2017b), the long cotyledonary axis (the ‘rope’) is the only intrinsic dispersal strategy that allows a seedling to establish some distance from the mother plant. In addition to reducing the impact of sibling competition, giant fruits likely sustain the establishment of young palms in a shady environment, until they are capable of producing the immensely long petioles that are characteristic of *Lodoicea* juveniles (Fig. 1c), permitting the leaf blade to reach the light of the canopy (Edwards *et al*., 2002). It is unlikely however that this alone drove the gigantism of *Lodoicea* since some smaller seeded relatives in Borasseae occur in habitats that are at least as shady.

Following past population reduction due to habitat loss, the double coconut has been over‐exploited as a medicinal plant and for souvenirs. *Lodoicea* is now rated as Endangered on the IUCN Red List of Threatened Species (Fleischer‐Dogley *et al*., 2011), and the remaining populations, amounting to *c*. 8000 wild mature individuals, are partly protected in national parks. The unique gigantism of the double coconut among its large‐seeded relatives reminds us that, even in the presence of genetic predisposition, time and contingency are necessary to evolve diverse structures and properties that will benefit humanity, be it by generating awe or medicinal remedies. The extinction from the wild of other palms of the syncarpous clade, such as *Latania*, *Medemia* or *Tahina*, is imminent (IUCN, 2020) and their loss could be equally, if not more detrimental to us in the long run, given the numerous services that they provide to nature and humanity (Dransfield *et al*., 2008).

## Author contributions

SB, RPB, SD, WE and WJB conceived the study. RPB performed field work, generated DNA data and aligned DNA sequences, LR, SB, HWP and PET generated seed size data, SB performed phylogenetic and molecular dating analyses with input from RPB and SD, SB performed biogeographic analyses with input from TLPC and WJB, and all other analyses with input from MSG, WLE and WJB. SB and WJB wrote the manuscript, with input from all co‐authors.

## Supporting information


**Fig. S1** Dated phylogeny of the syncarpous clade obtained by Bayesian analysis of two nuclear and three plastid regions.
**Fig. S2** Biogeographic hypotheses obtained with alternative models.
**Fig. S3** Rates of change in pyrene and seed size in the syncarpous clade.
**Fig. S4** Alternative measures of the rate of change in pyrene size in the syncarpous clade.

**Notes S1** Additional information on the biogeographical analyses.

**Table S1** Accession numbers and voucher information for the species included in the phylogenetic inferences.
**Table S2** Habitat, geographical distribution, plant size and inflorescence branching order of the sampled species.
**Table S3** Fruit, pyrene and seed measurements, including collection information.
**Table S4** Stepwise model selection procedure used to select the best linear model for each dataset.
Please note: Wiley Blackwell are not responsible for the content or functionality of any Supporting Information supplied by the authors. Any queries (other than missing material) should be directed to the *New Phytologist* Central Office.
