# Supplementary material for: On the origin of giant seeds: the macroevolution of the double coconut (Lodoicea maldivica) and its relatives (Borasseae, Arecaceae)
Source: New Phytol. 2020 Jul 29;228(3):1134–48. doi: 10.1111/nph.16750 (PMC7590125; doi:10.1111/nph.16750)
Supplement: Supplementary file 1 — Fig. S1 Dated phylogeny of the syncarpous clade obtained by Bayesian analysis of two nuclear and three plastid regions. Fig. S2 Biogeographic hypotheses obtained with alternative models. Fig. S3 Rates of change in pyrene and seed size in the syncarpous clade. Fig. S4 Alternative measures of the rate of change in pyrene size in the syncarpous clade. [file NPH-228-1134-s001.pdf]

New Phytologist Supporting Information
On the origin of giant seeds: the macroevolution of the double coconut (Lodoicea maldivica) and its relatives (Borasseae, Arecaceae).
Sidonie Bellot, Ross P. Bayton, Thomas L. P. Couvreur, Steven Dodsworth, Wolf L. Eiserhardt, Maïté S. Guignard, Hugh W. Pritchard, Lucy Roberts, Peter E. Toorop & William J. Baker
Article acceptance date: 29 May 2020

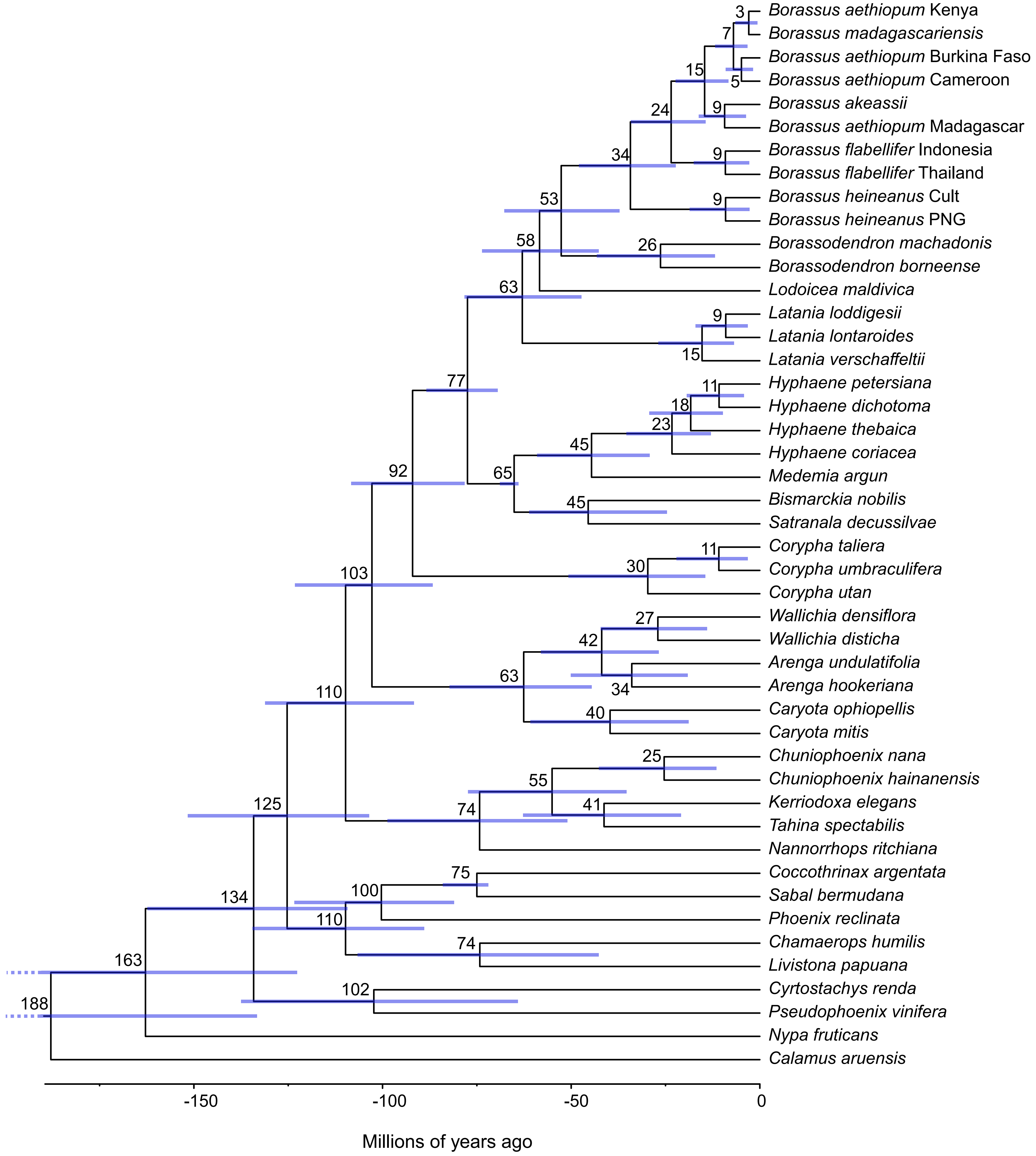

Figure S1. Dated phylogeny of the syncarpous clade obtained by Bayesian analysis of two nuclear and three plastid regions. Numbers at nodes are rounded ages in Ma. Blue bars represent 95% highest posterior density intervals of the ages.

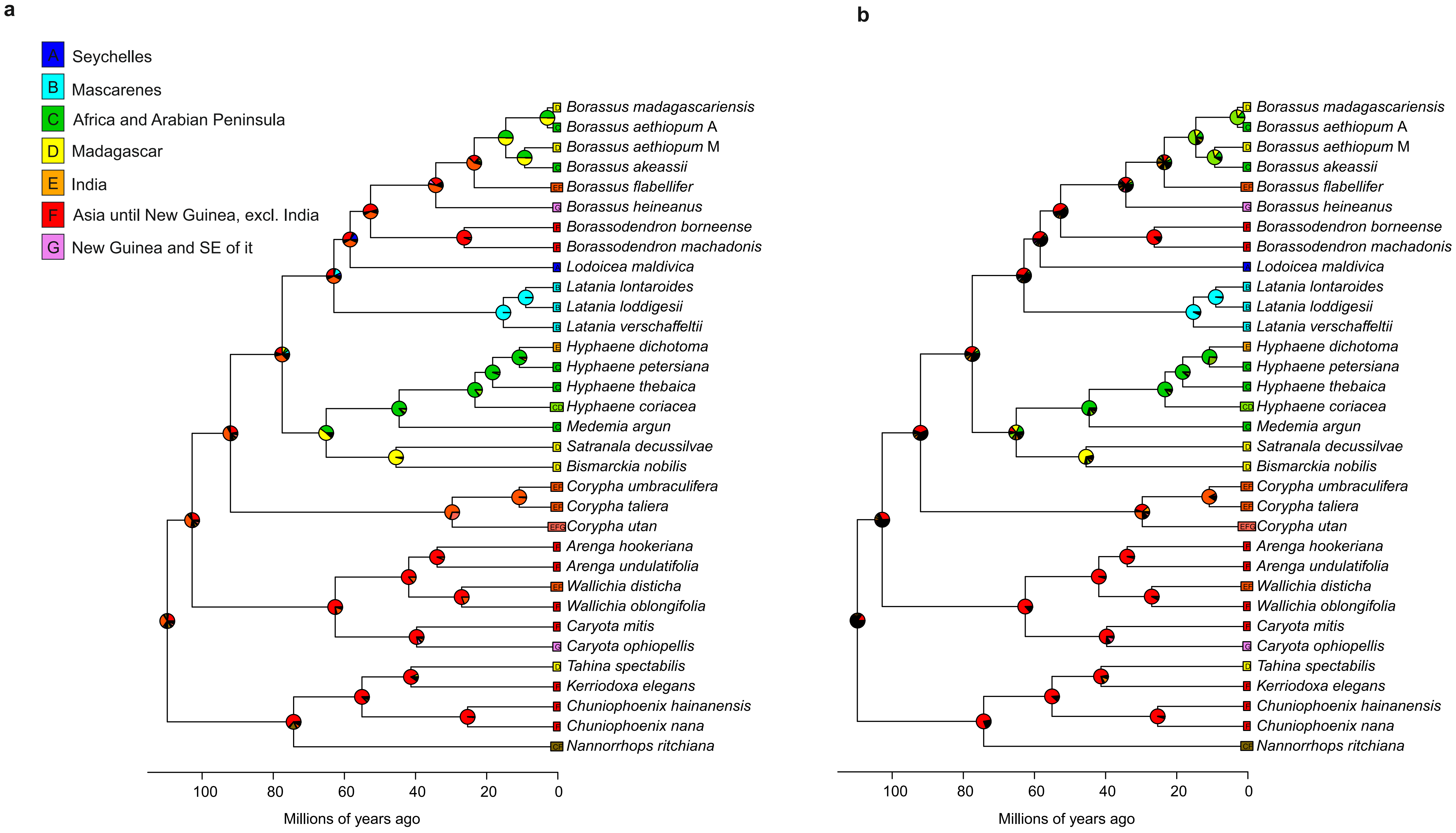

Figure S2. Biogeographic hypotheses obtained with alternative models. a. Ancestral range probabilities obtained using the BAYESAREALIKE model including founder-event speciation and the possibility of a “null” range (BJ*) implemented in BioGeoBEARS. b. Ancestral range probabilities obtained using the BAYESAREALIKE model including the possibility of a “null” range (B*) implemented in BioGeoBEARS.

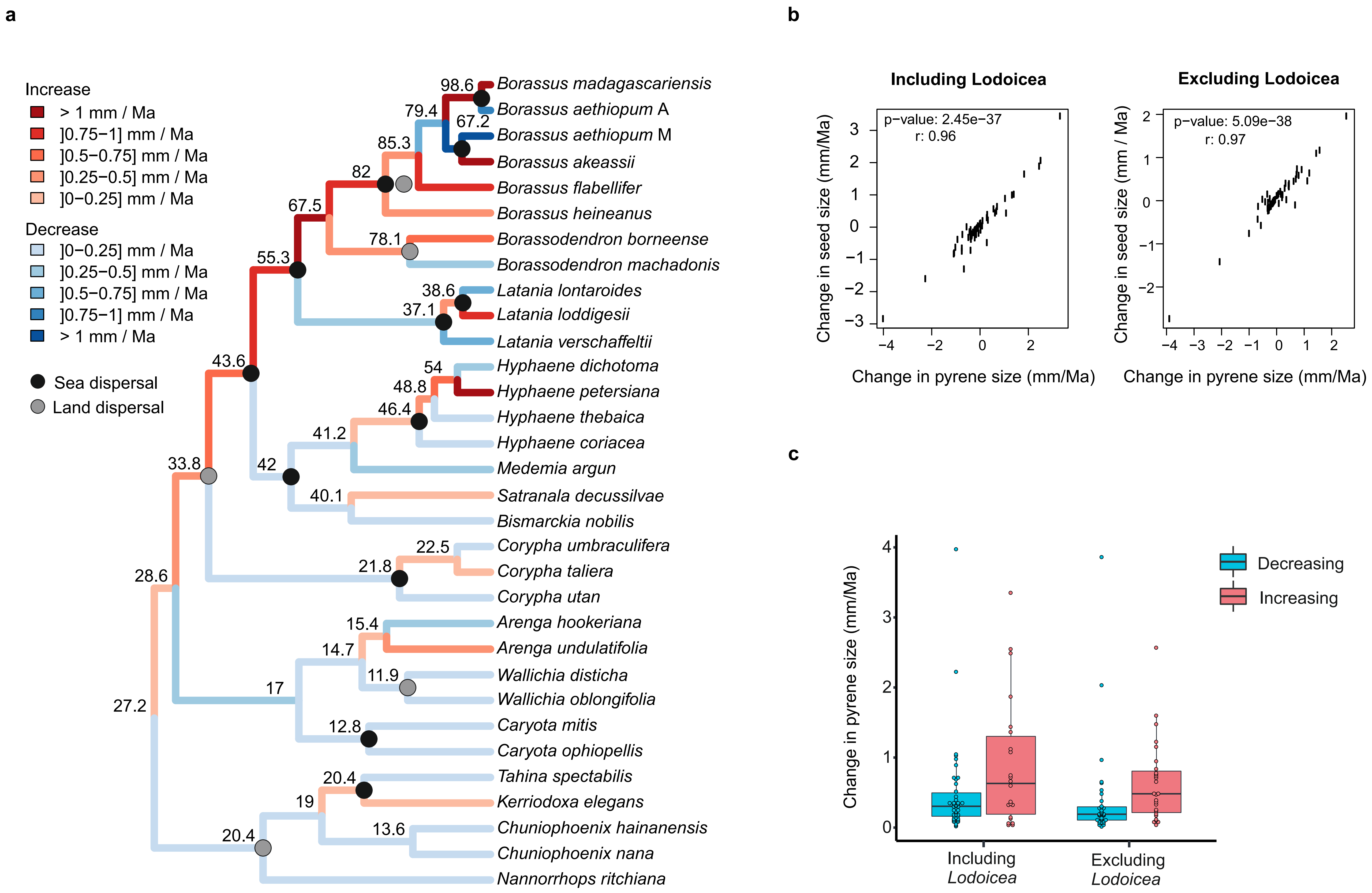

Figure S3. Rates of change in pyrene and seed size in the syncarpous clade. a. Ancestral pyrene sizes and rates of change in mm / Ma obtained when excluding *Lodoicea* from the analyses. b. Comparison of the rates of change in pyrene and seed sizes. c. Comparison of the magnitude of rates of increase and rates of decrease in pyrene size.

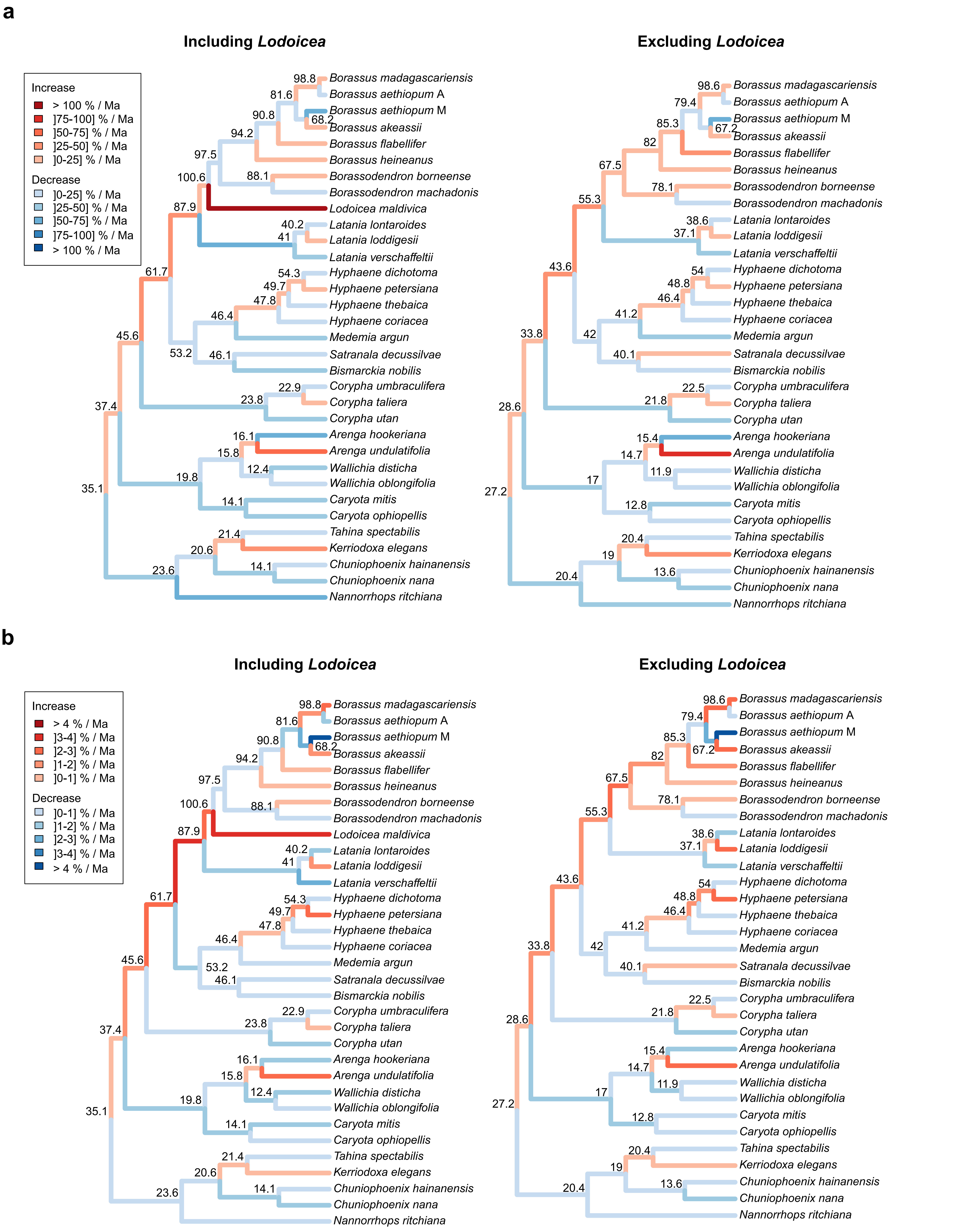

Figure S4. Alternative measures of the rate of change in pyrene size in the syncarpous clade. a. The rate of change between two nodes separated by a single branch is given as a percentage of the size of the parent node. b. The rate of change between two nodes separated by a single branch is given as a percentage of the size of the parent node divided by the time separating both nodes (in Ma).
