# Supplementary material for: On the origin of giant seeds: the macroevolution of the double coconut (Lodoicea maldivica) and its relatives (Borasseae, Arecaceae)
Source: New Phytol. 2020 Jul 29;228(3):1134–48. doi: 10.1111/nph.16750 (PMC7590125; doi:10.1111/nph.16750)
Supplement: Supplementary file 2 — Notes S1 Additional information on the biogeographical analyses. [file NPH-228-1134-s002.pdf]

**New Phytologist Supporting Information**

# On the origin of giant seeds: the macroevolution of the double coconut (Lodoicea maldivica) and its relatives (Borasseae, Arecaceae).

Sidonie Bellot, Ross P. Bayton, Thomas L. P. Couvreur, Steven Dodsworth, Wolf L. Eiserhardt, Maïté S. Guignard, Hugh W. Pritchard, Lucy Roberts, Peter E. Toorop & William J. Baker

Article acceptance date: 29 May 2020.

## Supplementary Notes S1. Additional information on the biogeographical analyses.

The biogeographic model with the smallest corrected Akaike Information Criterion (AICc) was BAYAREALIKE including founder-event speciation and the possibility of a "null" range (BJ*), with an AICc of 150.7, while the second best model was BAYAREALIKE including founder-event speciation and excluding the possibility of a "null" range (BJ), with an AICc of 152.1. Results of these models are presented in Figure S2a and Figure 3 respectively. Other models had an AICc superior to 161.8. Because the founder-event speciation parameter is controversial (Ree & Sanmartín, 2018), we also looked at the best model excluding it, which was BAYESAREALIKE including the possibility of a "null" range (B*), with an AICc of 162.4 (Figure S2b). The BJ* model recovered the ancestors of the syncarpous clade, the most recent common ancestor (MRCA) of Borasseae, the MRCA of Lataniinae and the MRCA of *Lodoicea* and *Borassus*+*Borassodendron* as all having Indo-Asian ranges (Fig. S2a), which would imply gene flow maintenance across the Neothetys ocean for millions of years (Chatterjee *et al.*, Gondwana Research, 2013, 23: 238–267), while the BJ and B* models recovered these ancestors as only being present in Asia (Fig. 3 and S2b). Other than this, the models only disagreed in the fact that the ancestor of African Borasseae was inferred to be in Africa *or* Madagascar by the BJ* (Fig. S2a) and BJ (Fig. 3) models but in Africa *and* Madagascar by the B* model (Fig. S2b), which seemed less probable given that by that time both landmasses had been separated for millions of years (Chatterjee *et al.*, 2013). We therefore decided to rely on the results obtained with the BJ model (Fig. 3).

## References

**Chatterjee S, Goswami A, Scotese CR**. **2013**. The longest voyage: Tectonic, magmatic, and paleoclimatic evolution of the Indian plate during its northward flight from Gondwana to Asia. *Gondwana Research* **23**: 238–267.

**Ree RH, Sanmartín I**. **2018**. Conceptual and statistical problems with the DEC+J model of founder-event speciation and its comparison with DEC via model selection. *Journal of Biogeography* **45**: 741–749.
